# Comparative Short-Term Outcomes of Double-Kissing Culotte and Culotte Techniques in Acute Coronary Syndrome from the Lower Silesia Culotte Bifurcation Registry

**DOI:** 10.3390/jcm13237392

**Published:** 2024-12-04

**Authors:** Mateusz Barycki, Adrian Włodarczak, Szymon Włodarczak, Maciej Pęcherzewski, Piotr Włodarczak, Artur Jastrzębski, Łukasz Furtan, Katarzyna Giniewicz, Adrian Doroszko, Piotr Rola, Maciej Lesiak

**Affiliations:** 1 Department of Cardiology, Provincial Specialized Hospital, 59-220 Legnica, Poland; lukas.furtan@gmail.com (Ł.F.); piotr.rola@gmail.com (P.R.); 2 Department of Cardiology, The Copper Health Centre (MCZ), 59-301 Lubin, Poland; wlodarczak.adrian@gmail.com (A.W.); wlodarczak.szy@gmail.com (S.W.); maciej999@gmail.com (M.P.); piotrwlodarczak123@gmail.com (P.W.); art.jastrzebski@gmail.com (A.J.); 3 Faculty of Medicine, Wroclaw University of Science and Technology, 50-981 Wroclaw, Poland; adrian.doroszko@pwr.edu.pl; 4 Independent Researcher, 50-030 Wrocław, Poland; katarzyna@giniewicz.it; 5 Department of Cardiology, Center for Heart Diseases, 4th Military Hospital, Faculty of Medicine, Wroclaw University of Science and Technology, 50-981 Wroclaw, Poland; 6 1st Department of Cardiology, University of Medical Sciences, 61-848 Poznan, Poland; maciej.lesiak@skpp.edu.pl

**Keywords:** acute coronary syndrome, percutaneous coronary intervention, endovascular procedures, bifurcation lesions, culotte technique

## Abstract

**Background/Objectives:** The double-kissing (DK) culotte technique is a modification of the culotte technique that employs initial kissing balloon inflation after first stent implantation. The DK culotte technique may improve strut apposition and procedural outcomes; however, data on its efficacy and safety remain limited. This study aimed to investigate the short-term outcomes of bifurcation percutaneous coronary intervention (PCI) using the DK culotte technique compared with those of the culotte technique in patients with acute coronary syndrome (ACS). **Methods:** This two-center, observational, retrospective study included patients with ACS. Out of 12,132 screened patients, 117 and 122 underwent DK culotte and culotte PCIs, respectively, with 117 and 57 patients remaining after propensity score matching. The primary endpoint was 1-year target lesion failure (TLF), which included cardiovascular death, target vessel myocardial infarction or clinically indicated target lesion revascularization (TLR). Secondary endpoints included major adverse cardiac events (MACEs) comprising myocardial infarction, cardiac death, and TLR; contrast medium amount (mL); and cumulative radiation dose (mGy). **Results:** At 1 year, TLF occurred in 7% and 12% of the DK culotte and culotte groups, respectively (*p* = 0.17). No significant differences were observed in MACEs between the groups (13% DK culotte vs. 19% culotte; *p* = 0.12). Additionally, the DK culotte technique did not cause higher contrast medium usage or cumulative radiation dosage. **Conclusions:** No statistically significant differences were found in TLF and MACE reduction between ACS patients treated with the DK culotte technique and the culotte technique. The observed trend favoring the DK culotte needs further validation in prospective studies.

## 1. Introduction

Bifurcation lesions remain a complex challenge for interventional cardiologists despite significant advances in the devices and techniques used in percutaneous coronary intervention (PCI). These lesions are relatively prevalent, accounting for 15–20% of all PCI cases [1]. The provisional stenting technique has recently shown more consistent results; however, due to the diversity and complexity of the anatomical subset in bifurcation lesions, a single stent strategy may cause the loss of functionally significant side branches, affecting short- and long-term outcomes [2,3,4,5]. This challenging scenario is associated with so-called ‘true’ bifurcations, where both the main branch (MB) and the side branch (SB) are significantly narrowed (>50% diameter stenosis), as well as situations that require implantation of a second stent following a provisional approach due to compromise of the side branch [6].

Two-stent techniques have been introduced into clinical practice to address these anatomical obstacles. Nevertheless, these techniques may result in a number of adverse characteristics, including the formation of multiple stent layers, neocarina formation, and incomplete bifurcation coverage due to stent malapposition, depending on the technique employed. This could significantly affect postoperative outcomes, particularly in a highly thrombotic subset of acute coronary syndrome (ACS). The optimal technique for two-stent PCI is currently a topic of ongoing debate, with increasing data available on this subject [7,8].

The culotte technique was introduced by Chevalier [9]. This technique involves the placement of a second stent through the cell of the initially placed stent toward the SB in the conventional method or the MB in the inverted method, leading to the formation of a short, two-layer stent overlap in the proximal part of the bifurcation and a short neocarina. The procedure is concluded with kissing balloon inflation (KBI) and a final proximal optimization technique (POT). This technique demonstrated a favorable safety profile and positive clinical outcomes [10,11]. However, a recent bench test study [12] indicates that this technique may carry the risk of pulling the multiple struts of the first implanted stent while placing the second stent, resulting in stent malapposition in the bifurcation area. This can result in incomplete stent coverage of the bifurcation, particularly when rewiring for the final KBI is performed incorrectly.

A recently introduced modification of the culotte technique, the double-kissing (DK) culotte, comprises an initial KBI immediately following the first stent implantation, followed by all traditional culotte technique steps. This initial KBI may help to preserve proper strut apposition in the bifurcation area throughout the procedure, thereby facilitating the procedure and reducing the risk of incomplete stent coverage of the bifurcation. There is a lack of data regarding the outcomes of the DK culotte technique, with only initial reports available [13,14].

Based on these preliminary insights, it can be assumed that the DK culotte technique may prove more effective than the standard culotte technique; however, clinical evaluation is still required. This study is one of the first to evaluate the performance of the DK culotte technique in clinical practice.

## 2. Materials and Methods

### 2.1. Study Population

**Figure 1:**
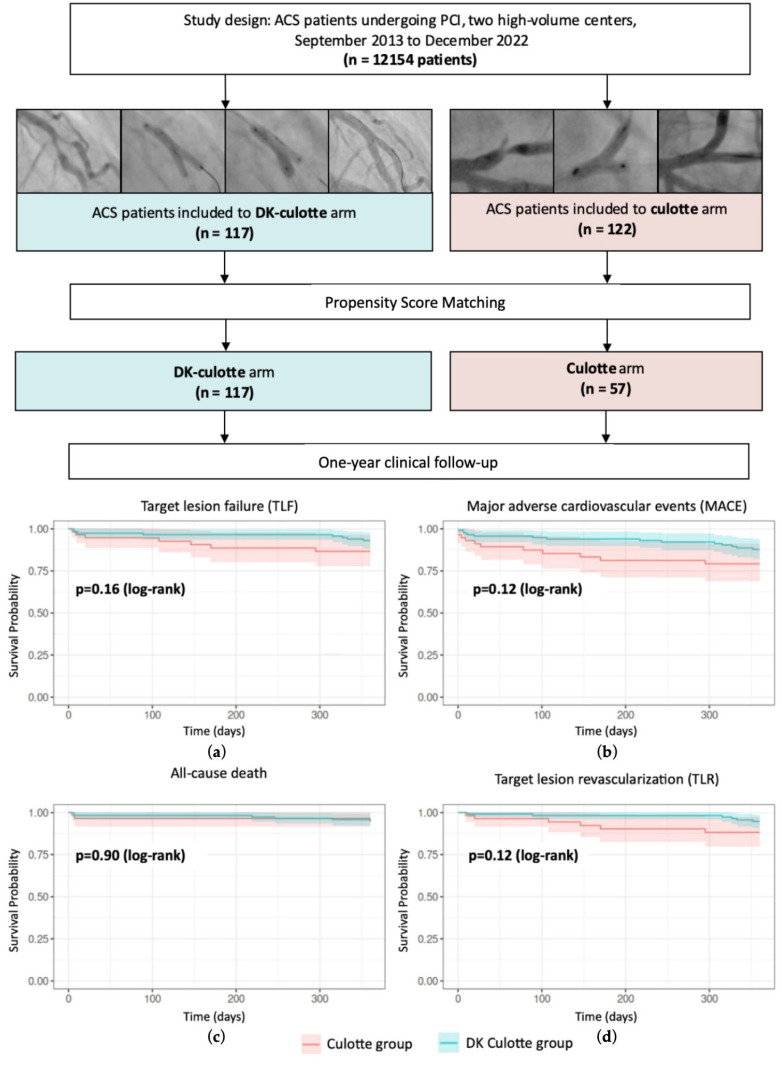
Central illustration. Comparative short-term outcomes of DK culotte and culotte techniques in acute coronary syndrome bifurcations: a two-center retrospective study (LSCBR registry). ACS—acute coronary syndrome; DK culotte—double-kissing culotte; LSCBR—The Lower Silesia Culotte Bifurcation Registry; MACE—major adverse cardiac event; PCI—percutaneous coronary intervention; TLF—target lesion failure; TLR—target lesion revascularization. (**a**) One-year Kaplan–Meier curves, demonstrating the TLF survival rates; (**b**) one-year Kaplan–Meier curves, demonstrating the MACE survival rates; (**c**) one-year Kaplan–Meier curves, demonstrating the all-cause death survival rates; (**d**) one-year Kaplan–Meier curves, demonstrating the TLR survival rates.

The Lower Silesia Culotte Bifurcation Registry (LSCBR) is a two-center retrospective analysis designed to evaluate the short-term outcomes of bifurcation PCI in patients with ACS using two different two-stent techniques, DK culotte and culotte. This study retrospectively analyzed subjects who underwent PCI in two high-volume cooperative Cardiac Departments in the Lower Silesia Region (Poland) from September 2013 to December 2022 during ACS. During these periods, all PCI data were screened for bifurcation lesions responsible for ACS requiring subsequent two-stent implantation. A total of 117 patients were assigned to the DK culotte group, and 122 patients were assigned to the control culotte group (see Figure 1).

Patients who qualified for the two-stent technique a priori and those who required a second stent as a bail-out strategy after the provisional approach were eligible for the registry. The decision to perform PCI was based on clinical indications, such as ongoing ischemia, significant angiographic coronary artery disease (CAD) that is suitable for PCI under the European Society of Cardiology recommendations, patient preference against alternative treatment options, or the decision of the Heart Team. The decision to perform PCI with the selected two-stent technique with second- and third-generation drug-eluting stents (DESs) was left to the interventional cardiologist’s discretion based on clinical and angiographic features. The analysis excluded patients who had undergone PCI with coronary stenting of the bifurcation lesion under investigation before the index procedure or suffered prehospital cardiac arrest. No vessel-related exclusion criteria were applied.

This study was approved by the Bioethics Committee of the Lower Silesian Medical Association (Poland) (01/BO/2023). All patients were fully informed of all treatment options and risks associated with PCI before signing written consent for the procedure. Due to the retrospective and observational nature of this study, the ethical committee approved the use of patient data collected in the registry to ensure that no identifying information or images were published. This study was conducted in accordance with the principles outlined in the Declaration of Helsinki. This study was registered in the ClinicalTrials.gov database under registration number NCT06284057.

### 2.2. Study Endpoints

This study’s primary endpoint was target lesion failure (TLF), which is a composite of cardiovascular (CV) death, target vessel myocardial infarction (TV-MI), or clinically driven target lesion revascularization (TLR) at the 1-year follow-up assessment [15]. The evaluated secondary endpoints were major adverse cardiac events (MACEs) (comprising MI, cardiac death, and TLR), TLR, all-cause mortality, stent thrombosis and stent restenosis. Additionally, this study assessed the prevalence of restenosis and thrombosis of the investigated lesion and health economic endpoints, such as contrast volume and cumulative radiation dose during PCI. TLR was defined as the repeat PCI of the target lesion or bypass surgery of the target vessel performed because of restenosis or other complications of the target lesion. The target lesion was the treated segment, including the 5 mm margin proximal and distal to the stent. MI was diagnosed following the Fourth Universal Definition of Myocardial Infarction [16]. The definition of stent thrombosis was derived from the Academic Research Consortium-2 Consensus Document [17].

### 2.3. Statistical Analysis

All statistical analyses were conducted using the R programming language by a statistician with expertise in medical analysis. The nonparametric two-sample Mann–Whitney *U* test was used for continuous variables. Fisher’s exact test for categorical variables was used to compare subjects between groups. The statistical significance cut-off point was set at 0.05, and lower *p*-values were considered significant. Data on one-year follow-up assessment results were available for all patients. Kaplan–Meier analysis was used to calculate the observed event rates at one year and the cumulative incidence of event-free survival, with comparisons made using the log-rank test. In order to address confounding factors between groups, a nearest neighbor matching method with replacement on logit of propensity score value was used as a selection method. Propensity score matching was performed using variables that showed a *p*-value < 0.05 in the baseline characteristics (diabetes mellitus, atrial fibrillation, left ventricular ejection fraction, intravascular imaging using OCT/IVUS, and final POT inflation), as well as the bifurcation lesion involving the left main artery, which was included due to its clinical significance. The balance of covariates post-matching was assessed to confirm the effectiveness of the matching procedure. For normal continuous random variables, a standardized mean difference was used, using Hedges’ g* estimate and confidence intervals using the method of Cousineau and Goulet-Pelletier. For non-normal continuous variables, a Glass rank biserial correlation coefficient r is reported and for discrete covariates, and Cramer’s V effect size has been reported, both with confidence intervals calculated using BCa bootstrap method. Additionally, a multivariate analysis including variables potentially associated with the outcomes of TLF and MACE was performed using the Cox proportional hazards model.

## 3. Results

### 3.1. Patient Characteristics

Table 1 shows all patient characteristic data before and after propensity score matching (PSM). The average age of patients in both groups was similar, with a predominance of males in each group (DK culotte group: 75% and culotte group: 70%). Before applying PSM, a significant difference was observed in the prevalence of unstable angina, with a higher rate in the DK culotte group (48% vs. 35%, *p* = 0.050). Among all comorbidities, diabetes mellitus type 2 and atrial fibrillation were observed less frequently in the DK culotte group compared with the culotte group before PSM (31% vs. 48%, *p* = 0.01 for diabetes mellitus type 2 and 11% vs. 22%, *p* = 0.03 for atrial fibrillation). The prevalence of lipidemic disorders was similar in both groups. No significant differences in the discharge regimens of antiplatelet and anticoagulant medications were found.

### 3.2. Lesions and Procedural Characteristics

Table 2 shows detailed procedural-related data. The complexity of CAD, as assessed using the SYNTAX score, was similar across both groups (DK culotte median: 15 points vs. culotte median: 15 points). However, the DK culotte group had lower risk of mortality, as evaluated using the Logistic SYNTAX score and SYNTAX II before PSM (2.9 points vs. 4.2 points and 28.6 points vs. 32.95 points, respectively), with these differences being balanced after PSM. The use of the two-stent technique as a bail-out strategy was relatively infrequent, accounting for 8% and 9% of PCIs in the DK culotte and culotte groups, respectively. The most frequently used technique was the inverted stenting technique, which involves stenting the SB first in both groups. The DK culotte resulted in a 100% success rate for the final KBI procedure. After PSM, the differences in the incidence of final POT between the groups were balanced. Both groups demonstrated low levels of image-guided PCI.

### 3.3. Clinical Outcomes

**Figure 2:**
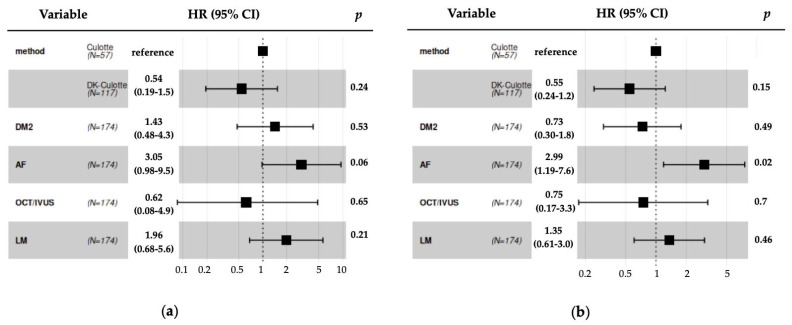
(**a**)—Multivariate Cox proportional hazards analysis for target lesion failure after propensity score matching. (**b**)—Multivariate Cox proportional hazards analysis for major adverse cardiac events after propensity score matching. AF: atrial fibrillation; CI: confidence interval; DM2: diabetes mellitus type 2; HR: hazard ratio; IVUS: intravascular ultrasound; LM: left main; OCT: optical coherence tomography.

Table 3 presents the hazard ratio and *p*-value for the univariate Cox model, comparing survival functions between two intervention methods. Figure 2 presents the multivariate Cox model analysis for TLF and MACE after PSM. The analysis of the TLF rate at the 1-year follow-up did not show a statistically significant difference in outcomes between the two study cohorts, despite numerical differences in favor of the DK culotte group. This follow-up point revealed TLF in 7% and 12% of patients in the DK culotte group and culotte groups, respectively (*p* = 0.17). None of the patients who received bail-out strategy treatment in either group experienced the primary endpoint. The incidence of stent restenosis was not significantly different between the two groups (3% vs. 7%, *p* = 0.269). Figure 1 presents the Kaplan–Meier curves, which demonstrate the TLF, MACE, all cause mortality and TLR survival rates after PSM. The analysis of secondary endpoints for MACEs also did not reach statistical significance, despite a favorable trend for the DK culotte (13% vs. 19%; *p* = 0.12). In the multivariate Cox regression analysis, atrial fibrillation was identified as a significant predictor of adverse outcomes, with a hazard ratio for MACE of 2.99 (95% CI: 1.19-7.6; *p* = 0.02).

## 4. Discussion

To the best of our knowledge, this study is the first to investigate clinical outcomes following the treatment of bifurcation lesions using two different two-stent techniques, DK culotte versus culotte, in a real-life ACS cohort.

The study results showed no statistically significant difference between the two-stent techniques in terms of 1-year follow-up assessment results. However, there was a trend towards a lower incidence of 1-year TLF and MACEs in the DK culotte group compared with the classic culotte group, which was primarily due to a lower number of TLR events. However, after propensity score matching, differences were less pronounced. Additionally, no significant differences in contrast volume or radiation dose were found between the two evaluated stenting techniques.

It is crucial to acknowledge that the lengthy recruitment period reflected in the registry permits us to observe shifts in operator practices. Initially, the culotte technique was the most prevalent, accompanied by a low rate of pre-POT and final POT inflation. In subsequent years, the use of the DK culotte technique became more prevalent, and the frequency of pre-POT and final POT inflations increased in the culotte procedures. The low rate of POT inflations in the culotte group during the early years may have influenced the final events in this group. However, the favorable trend for the DK culotte technique persisted even after correcting for the effect of the final POT using propensity score matching.

According to the revascularization guidelines [18], provisional stenting is the most applicable solution in most bifurcation cases. However, operators may need to consider a two-stent technique in more complex coronary bifurcation anatomies, especially in the ACS subset where preserving good flow to the SB may be crucial to achieve a favorable clinical outcome and potentially reduce ischemic burden. Several anatomical features may encourage this approach. Procedural factors that influence the adoption of up-front two-stent strategies include a large SB (≥2.75 mm) with a long ostial lesion (at least >5 mm), anticipated difficulty in accessing the SB after MB stenting, and true bifurcation lesions [19,20].

The bifurcation consensus document [3] by the European Bifurcation Club listed three main two-stent techniques: “T/TAP”, “culotte”, and “DK-crush”. Since the development of the DK-crush technique by Chen et al. [21], several landmark trials have demonstrated the safety and efficacy of this technique, particularly compared with classic crush. A modification to the classic crush technique, involving the addition of an extra KBI, has significantly improved the long-term results of the DK-crush technique. Various studies have revealed that KBI is a crucial aspect of bifurcation management.

Since its introduction to clinical practice [9], the technically demanding bifurcation stenting technique known as culotte has become widely used because of its satisfactory safety and efficiency [10,22]. However, some data indicate that the DK-crush technique may provide benefits over culotte [23,24]. Recent bench tests have demonstrated that the culotte technique carries the risk of dislodging multiple struts of the first implanted stent away from the branch wall [12]. This misalignment poses a risk of rewiring under malapposed struts for the final KBI, which may ultimately result in incomplete stent coverage of the bifurcation. The initial KBI in the DK culotte technique ensures proper apposition of the first implanted stent struts at the bifurcation level, facilitating the correct performance of the final KBI.

This modification of the culotte technique is promising for improving outcomes. However, clinical studies that investigate the significant effects and long-term outcomes of adding KBI to the culotte technique remain lacking.

It is important to be aware of the limitations of this study in the interpretation of the results. The primary limitation of this study is its observational nature, which resulted in heterogeneity among the study groups. This was particularly evident in the cases involving type 2 diabetes mellitus, atrial fibrillation, intravascular imaging with OCT/IVUS, and the complexity of the coronary pathology assessed using the SYNTAX Logistic score and SYNTAX II. This heterogeneity may be a significant limitation to the interpretation of the results and their generalizability. Additionally, statistical significance was not achieved despite a clear trend favoring the DK culotte technique, which is possibly due to the small size of the study group. Various guidelines were in place regarding dual antiplatelet and antithrombotic therapy from 2013 to 2022 (registry scope), which may have influenced adverse cardiovascular events in patients undergoing the two-stent technique. The absence of quantitative coronary angiography data limits the depth of angiographic analysis, potentially impacting the precision of our findings regarding bifurcation lesion characteristics. However, given the lack of information on the clinical performance of the DK culotte technique, the data provided are still valuable.

The results of this two-center retrospective analysis are the first available data on the short-term prognosis of the DK culotte method compared with the culotte method. Adding the KBI step is a safe method of modifying the culotte technique that facilitates the performance of the final KBI procedure without resulting in excessive contrast or radiation dose usage. Further follow-up and prospective studies are recommended to distinguish the benefits of the DK culotte from the traditional culotte technique, particularly to determine any long-term advantages.

## 5. Conclusions

The results indicate a trend towards better clinical outcomes with the double-kissing culotte technique without increased procedural risks; however, no statistically significant differences were found in target lesion failure and major adverse cardiac events reduction between the double-kissing culotte and culotte techniques. These findings do not provide sufficient evidence to conclude a definitive benefit of the double-kissing culotte technique, and further validation in prospective studies is needed.

## Figures and Tables

**Table 1:** Baseline patient characteristics.

	*Without Propensity Score Matching*				*After Propensity Score Matching*			
	DK Culotte N = 117	Culotte N = 122	Effect Size (95% CI)	*p*	DK Culotte N = 117	Culotte N = 57	Effect Size (95% CI)	*p*
Age ± SD, years Male sex, n (%)	67 ± 9.4	66 ± 9.1	g* = −0.05 (−0.30−0.21)	0.71	67 ± 9.4	65 ± 8.1	g* = −0.22 (−0.54−0.10)	0.17
	88 (75%)	85 (70%)	V = 0.06 (0−0.20)	0.39	88 (75%)	42 (74%)	V = 0.02 (0−0.17)	0.85
**Clinical presentation** Unstable angina, n (%)			V = 0.13 (0−0.26)	0.13			V = 0.09 (0−0.24)	0.54
	56 (48%)	43 (35%)			56 (48%)	24 (42%)		
NSTEMI, n (%)	41 (35%)	55 (45%)			41 (35%)	25 (44%)		
STEMI, n (%)	20 (17%)	24 (20%)			20 (17%)	8 (14%)		
**Clinical history**								
Diabetes mellitus type 2, n (%)	36 (31%)	58 (48%)	V = 0.17 (0.08−0.30)	0.01	36 (31%)	23 (40%)	V = 0.10 (0−0.26)	0.24
Insulin-dependent Diabetes, n (%) Hypertension, n (%)	10 (9%)	12 (10%)	V = 0.02 (0−0.16)	0.82	10 (9%)	3 (5%)	V = 0.06 (0−0.22)	0.55
	94 (80%)	103 (84%)	V = 0.05 (0−0.19)	0.50	94 (80%)	49 (86%)	V = 0.07 (0−0.23)	0.41
Hyperlipidemia, n (%)	98 (85%)	91 (75%)	V = 0.11 (0−0.25)	0.11	98 (85%)	41 (72%)	V = 0.14 (0−0.30)	0.07
Atrial Fibrillation, n (%)	13 (11%)	27 (22%)	V = 0.12 (0.07−0.28)	0.03	13 (11%)	11 (19%)	V = 0.11 (0−0.27)	0.16
Current smoking, n (%)	36 (31%)	45 (37%)	V = 0.07 (0−0.20)	0.34	36 (31%)	20 (35%)	V = 0.04 (0−0.21)	0.61
COPD/Asthma Bronchial, n (%)	5 (4%)	14 (12%)	V = 0.13 (0−0.27)	0.05	5 (4%)	4 (7%)	V = 0.06 (0−0.22)	0.48
Previous PCI, n (%)	37 (32%)	42 (34%)	V = 0.03 (0−0.17)	0.68	37 (32%)	20 (35%)	V = 0.04 (0−0.20)	0.73
Previous MI, n (%)	34 (29%)	30 (25%)	V = 0.05 (0−0.19)	0.47	34 (29%)	13 (23%)	V = 0.07 (0−0.23)	0.47
Previous CABG, n (%)	3 (3%)	7 (6%)	V = 0.08 (0−0.22)	0.33	3 (3%)	2 (4%)	V = 0.03 (0−0.19)	0.66
Dialysis, n (%)	2 (2%)	1 (1%)	V = 0.04 (0−0.18)	0.62	2 (2%)	1 (2%)	V = 0.002 (0−1)	1
Ischemic stroke/TIA history, n (%)	4 (3%)	10 (8%)	V = 0.10 (0−0.24)	0.17	4 (3%)	5 (9%)	V = 0.11 (0−0.27)	0.16
LVEF, %, median (IQR)	56 (45–63)	55 (40–60)	r = −0.15 (−0.29−0.01)	0.04	56 (45–63)	55 (44−64)	r = −0.004 (−0.21−0.19)	0.96
**Laboratory values**								
Total cholesterol, mmol/L, median (IQR)	4.6 (3.9–5.5)	4.6 (3.95–5.85)	r = 0.08 (−0.07−0.21)	0.32	4.6 (3.9–5.5)	4.3 (3.6−5.5)	r = −0.08 (−0.26−0.12)	0.41
LDL, mmol/L, median (IQR)	2.6 (1.9–3.4)	2.7 (1.9–3.63)	r = 0.08 (−0.08−0.24)	0.28	2.6 (1.9–3.4)	2.3 (1.8−3.4)	r = −0.05 (−0.24−0.14)	0.63
HDL, mmol/L, median (IQR)	1.2 (1.0–1.4)	1.2 (1.0–1.5)	r = −0.004 (−0.15−0.14)	0.95	1.2 (1.0–1.4)	1.3 (1.1−1.5)	r = 0.01 (−0.16−0.20)	0.91
Hemoglobin, baseline, g/dL, median (IQR)	14.1 (13.1–15.1)	14.2 (13.1–14.9)	r = 0.02 (−0.14−0.16)	0.81	14.1 (13.1–15.1)	14.5 (13.2−15)	r = 0.09 (−0.08−0.26)	0.36
Creatine, µmol/l, median (IQR)	82.0 (68.8–96.5)	85.7 (72.0–100.0)	r = 0.08 (−0.07−0.23)	0.28	82.0 (68.8–96.5)	81.3 (68.1−97)	r = −0.02 (−0.22−0.17)	0.83
**Antiplatelets and anticoagulants at discharge**								
ASA, n (%)	117 (100%)	122 (100%)		N/A	117 (100%)	57 (100%)		N/A
Clopidorel, n (%)	68 (58%)	83 (68%)	V = 0.10 (0−0.24)	0.14	68 (58%)	31 (54%)	V = 0.04 (0−0.20)	0.75
Tikagrelor, n (%)	43 (37%)	38 (31%)	V = 0.06 (0−0.20)	0.41	43 (37%)	25 (44%)	V = 0.07 (0−0.23)	0.41
Prasugrel, n (%)	4 (3%)	0 (0%)	V = 0.13 (0−0.27)	0.06	4 (3%)	0 (0%)	V = 0.11 (0−0.27)	0.31
NOAC, n (%)	13 (11%)	20 (16%)	V = 0.08 (0−0.21)	0.26	13 (11%)	9 (16%)	V = 0.07 (0−0.23)	0.47
VKA, n (%)	3 (3%)	6 (5%)	V = 0.06 (0−0.20)	0.50	3 (3%)	2 (4%)	V = 0.03 (0−0.19)	0.66

ASA: acetylosalicylic acid; CABG: coronary artery bypass graft; CI: confidence interval; COPD: chronic obstructive pulmonary disease; DK: double kiss; g*: Hedges’ g* estimate; HDL: high-density lipoprotein; IQR: interquartile range; LDL: low-density lipoprotein; LVEF: left ventricular ejection fraction; MI: myocardial infarction; N/A: not applicable; NOAC: novel oral anticoagulant; NSTEMI: non-ST-elevation myocardial infarction; PCI: percutaneous coronary intervention; r: Glass rank biserial correlation coefficient r; SD: standard deviation; STEMI: ST-elevation myocardial infarction; TIA: transient ischemic attack; V: Cramer’s V effect size; VKA: vitamin K antagonist.

**Table 2:** Baseline angiographic and procedural characteristics.

	*Without Propensity Score Matching*				*After Propensity Score Matching*			
	DK Culotte N = 117	Culotte N = 122	Effect Size (95% CI)	*p*	DK Culotte N = 117	Culotte N = 57	Effect Size (95% CI)	*p*
**Vessel and clinical assessment:** SYNTAX Score I, median (IQR)								
	15 (11–21)	16.5 (12–26)	r = 0.12 (−0.03−0.26)	0.12	15 (11–21)	15 (12−20.5)	r = 0.02 (−0.16−0.20)	0.84
Logistic SYNTAX Score, median (IQR) PCI SYNTAX Score II, median (IQR)	2.9 (1.5−5.5)	4.2 (1.6–11.0)	r = 0.16 (−0.01−0.29)	0.04	2.9 (1.5−5.5)	2.6 (1.3−5.8)	r = −0.05 (−0.22−0.15)	0.60
	28.6 (22.1–39.3)	32.95 (25.4–44.68)	r = 0.18 (0.04−0.33)	0.02	28.6 (22.1–39.3)	30.1 (22.2−39)	r = 0.05 (−0.14−0.24)	0.61
**Bifurcation lesion location:**			V = 0.07 (0−0.21)	0.14			V = 0.02 (0−0.17)	0.87
LM, n (%)	47 (40%)	41 (34%)			47 (40%)	24 (42%)		
non-LM (LAD/D), n (%)	48 (41%)	43 (35%)			48 (41%)	19 (33%)		
non-LM (Cx/OM), n (%)	17 (15%)	31 (25%)			17 (15%)	10 (18%)		
non-LM (RCA/PLA), n (%)	5 (4%)	7 (6%)			5 (4%)	4 (7%)		
**PCI access:**			V = 0.01 (0−0.14)	0.72			V = 0.01 (0−0.12)	0.92
Femoral access, n (%)	18 (15%)	20 (16%)			18 (15%)	9 (16%)		
Radial access, n (%)	99 (85%)	102 (84%)			99 (85%)	49 (86%)		
**Procedure characteristics:**								
Bail out two stent strategy, n (%)	9 (8%)	9 (7%)	V = 0.01 (0−0.12)	0.93	9 (8%)	5 (9%)	V = 0.02 (0−0.17)	0.78
Side branch stent diameter, mm median (IQR)	3.0 (2.75–3.0)	3.0 (2.5–3.5)	r = 0 (−0.14−0.15)	0.99	3.0 (2.75–3.0)	3 (2.8−3.50)	r = 0.09 (−0.10−0.25)	0.33
Side branch stent length, mm median (IQR)	20 (18–26)	22 (18–28)	r = 0.11 (−0.04−0.25)	0.16	20 (18–26)	22 (18−26)	r = 0.06 (−0.13−0.24)	0.49
Main branch stent diameter, mm median (IQR)	4.0 (3.0–3.5)	4.0 (3.0–3.5)	r = −0.07 (−0.21, 0.06)	0.36	4.0 (3.0–3.5)	3.5 (3−3.5)	r = 0.03 (−0.14−0.21)	0.72
Main branch stent length, mm median (IQR)	25 (18–26)	26 (18–30)	r = 0.04 (−0.10−0.18)	0.62	25 (18–26)	26 (18−28)	r = −0.02 (−0.21−0.17)	0.81
Stent to the side branch first, n (%)	99 (85%)	103 (84%)	V = 0.003 (0−0.08)	0.97	99 (85%)	45 (79%)	V = 0.07 (0−0.23)	0.40
Side branch predilatation, n (%)	97 (83%)	102 (84%)	V = 1 (0.88−1)	0.88	97 (83%)	46 (81%)	V = 0.12 (0.10−0.67)	0.58
Main branch predilatation, n (%)	85 (73%)	106 (87%)	V = 0.18 (0.08−0.31)	0.01	85 (73%)	51 (90%)	V = 0.19 (0.09−0.35)	0.011
Pre POT, n (%)	105 (90%)	55 (45%)	V = 0.48 (0.35−0.61)	<0.001	105 (90%)	26 (46%)	V = 0.48 (0.34−0.63)	<0.001
KB after the first stent implantation, n (%)	117 (100%)	0 (0.0%)	V = 1 (0.88−1)	<0.001	117 (100%)	0 (0%)	V = 1 (0.86−1)	<0.001
KB after the second stent implantation, n (%)	117 (100%)	119 (98%)	V = 0.11 (0−0.246)	N/A	117 (100%)	57 (100%)		N/A
Final POT, n (%)	111 (95%)	98 (80%)	V = 0.22 (0.11−0.35)	0.01	111 (95%)	56 (98%)	V = 0.08 (0−0.24)	0.43
IVUS/OCT imaging, n (%)	16 (14%)	5 (4%)	V = 0.16 (0.07−0.30)	0.01	16 (14%)	4 (7%)	V = 0.11 (0−0.27)	0.22
Rotablation, n (%)	5 (4%)	6 (5%)	V = 0.02 (0−0.15)	0.81	5 (4%)	5 (9%)	V = 0.09 (0−0.25)	0.30
Intravascular lithotripsy, n (%)	2 (2%)	1 (1%)	V = 0.04 (0−0.18)	0.53	2 (2%)	1 (2%)	V = 0.002 (0−1)	1
GP IIb/IIIa use, n (%)	2 (2%)	11 (9%)	V = 0.16 (0.07−0.30)	0.01	2 (2%)	1 (2%)	V = 0.002 (0−1)	1
Radiation dose (mGy) median (IQR)	1868 (1178–2891)	2114.5 (1372.5–3265)	r = 0.11 (−0.03−0.26)	0.13	1868 (1178–2891)	1999 (1258−2717)	r = 0.03 (−0.15−0.22)	0.78
Contrast media amount (mL) median (IQR)	220 (170–280)	230 (200–270)	r = 0.09 (−0.06−0.25)	0.21	220 (170–280)	237.1 ± 70.9	g* = 0.13 (−0.19−0.45)	0.44

CI: confidence interval; Cx: circumflex; D: diagonal; DK: double kissing; GP: glycoprotein; IQR: interquartile range; IVUS: intravascular ultrasound; KB: kissing balloon; LAD: left anterior descending; LM: left main; OCT: optical coherence tomography; OM: obtuse marginal; PCI: percutaneous coronary intervention; N/A: not applicable; PLA: posterolateral artery; POT: proximal optimization technique; r: Glass rank biserial correlation coefficient r; RCA: right coronary artery; RVA: right ventricular artery; SD: standard deviation; V: Cramer’s V effect size.

**Table 3:** Outcomes from univariate Cox proportional hazards model after propensity score matching.

	DK Culotte Group N = 117	Culotte Group N = 57	HR (95% CI)	*p*
**1-year follow-up primary outcome:** Primary outcome: Target lesion failure (cardiac death, target vessel myocardial infarct, target lesion revascularisation), n (%)				
	8 (7%)	7 (12%)	0.49 (0.18–1.36)	0.17
**1-year follow-up secondary outcome:** Principal secondary outcome: MACE (myocardial infarct, cardiac death, target lesion revascularization), n (%)				
	15 (13%)	11 (19%)	0.54 (0.24–1.18)	0.12
Target lesion revascularization, n (%)	6 (5%)	6 (11%)	0.42 (0.14–1.30)	0.13
All-cause mortality, n (%)	6 (5%)	3 (5%)	0.91 (0.23–3.65)	0.90
Stent thrombosis, n (%)	4 (3%)	2 (4%)	0.70 (0.12–4.19)	0.69
Stent restenosis, n (%)	4 (3%)	4 (7%)	0.42 (0.11–1.67)	0.22

CI: confidence interval; DK: double kissing; HR: hazard ratio; MACE: major adverse cardiac event.

## Data Availability

The information and data of the study population were extracted from the Hospital Information System. The datasets are not publicly available because the individual privacy of the patients should be protected. However, the data are available from the corresponding author upon reasonable request.

## References

[1] Steigen T.K., Maeng M., Wiseth R., Erglis A., Kumsars I., Narbute I., Gunnes P., Mannsverk J., Meyerdierks O., Rotevatn S. (2006). Randomized study on simple versus complex stenting of coronary artery bifurcation lesions: The Nordic bifurcation study. Circulation.

[2] Elwany M., Palma G.D., Cortese B. (2018). Treatment of coronary bifurcation lesions: Current knowledge and future perspectives. Future Cardiol..

[3] Albiero R., Burzotta F., Lassen J.F., Lefèvre T., Banning A.P., Chatzizisis Y.S., Johnson T.W., Ferenc M., Pan M., Daremont O. (2022). Treatment of coronary bifurcation lesions, part I: Implanting the first stent in the provisional pathway. The 16th expert consensus document of the European Bifurcation Club. EuroIntervention.

[4] Rigatelli G., Zuin M., Gianese F., Adami D., Carraro M., Roncon L. (2022). Single versus Double Stenting in NSTEMI Patients with Complex Left Main Bifurcation Disease. J. Clin. Med..

[5] Krittanawong C., Virk H.U.H., Qadeer Y.K., Irshad U., Wang Z., Alam M., Sharma S. (2023). Clinical Outcomes Following Bifurcation Techniques for Percutaneous Coronary Intervention. J. Clin. Med..

[6] Lassen J.F., Albiero R., Johnson T.W., Burzotta F., Lefèvre T., Iles T.L., Pan M., Banning A.P., Chatzizisis Y.S., Ferenc M. (2022). Treatment of coronary bifurcation lesions, part II: Implanting two stents. The 16th expert consensus document of the European Bifurcation Club. EuroIntervention.

[7] Di Gioia G., Sonck J., Ferenc M., Chen S.L., Colaiori I., Gallinoro E., Mizukami T., Kodeboina M., Nagumo S., Franco D. (2022). Clinical Outcomes Following Coronary Bifurcation PCI Techniques: A Systematic Review and Network Meta-Analysis Comprising 5,711 Patients. JACC Cardiovasc. Interv..

[8] Bujak K., Verardi F.M., Arevalos V., Gabani R., Spione F., Rajwa P., Milasinovic D., Stankovic G., Gasior M., Sabaté M. (2023). Clinical outcomes following different stenting techniques for coronary bifurcation lesions: A systematic review and network meta-analysis of randomised controlled trials. EuroIntervention.

[9] Chevalier B., Glatt B., Royer T., Guyon P. (1998). Placement of coronary stents in bifurcation lesions by the “culotte” technique. Am. J. Cardiol..

[10] Ferenc M., Gick M., Comberg T., Rothe J., Valina C., Toma A., Löffelhardt N., Hochholzer W., Riede F., Kienzle R.P. (2016). Culotte stenting vs. TAP stenting for treatment of de-novo coronary bifurcation lesions with the need for side-branch stenting: The Bifurcations Bad Krozingen (BBK) II angiographic trial. Eur. Heart J..

[11] Walsh S.J., Hanratty C.G., Watkins S., Oldroyd K.G., Mulvihill N.T., Hensey M., Chase A., Smith D., Cruden N., Spratt J.C. (2018). Culotte stenting for coronary bifurcation lesions with 2nd and 3rd generation everolimus-eluting stents: The CELTIC Bifurcation Study. EuroIntervention.

[12] Toth G.G., Sasi V., Franco D., Prassl A.J., Di Serafino L., Ng J.C.K., Szanto G., Schneller L., Ang H.Y., Plank G. (2020). Double-kissing culotte technique for coronary bifurcation stenting. EuroIntervention.

[13] Barycki M., Rola P., Włodarczak A., Włodarczak S., Pęcherzewski M., Włodarczak P., Jastrzębski A., Furtan Ł., Giniewicz A., Doroszko A. (2024). One-year comparative outcomes of DK culotte and culotte techniques in left main bifurcation in acute coronary syndrome: Sub-study of the Lower Silesia culotte Bifurcation Registry. Kardiol. Pol..

[14] Tu S., Zhang L., Tian Q., Hu F., Wang Y., Chen L. (2024). Five-year outcomes of double kissing mini-culotte stenting vs. mini-culotte stenting using drug-eluting stents for the treatment of true coronary bifurcation lesions. Front. Cardiovasc. Med..

[15] Lunardi M., Louvard Y., Lefèvre T., Stankovic G., Burzotta F., Kassab G.S., Lassen J.F., Darremont O., Garg S., Koo B.K. (2023). Definitions and Standardized Endpoints for Treatment of Coronary Bifurcations. EuroIntervention.

[16] Thygesen K., Alpert J.S., Jaffe A.S., Chaitman B.R., Bax J.J., Morrow D.A., White H.D. (2018). Executive Group on behalf of the Joint European Society of Cardiology (ESC)/American College of Cardiology (ACC)/American Heart Association (AHA)/World Heart Federation (WHF) Task Force for the Universal Definition of Myocardial Infarction. Fourth universal definition of myocardial infarction. J. Am. Coll. Cardiol..

[17] Garcia-Garcia H.M., McFadden E.P., Farb A., Mehran R., Stone G.W., Spertus J., Onuma Y., Morel M.A., van Es G.A., Zuckerman B. (2018). Standardized End Point Definitions for Coronary Intervention Trials: The Academic Research Consortium-2 Consensus Document. Circulation.

[18] Neumann F.J., Sousa-Uva M., Ahlsson A., Alfonso F., Banning A.P., Benedetto U., Byrne R.A., Collet J.P., Falk V., Head S.J. (2019). 2018 ESC/EACTS Guidelines on myocardial revascularization. EuroIntervention.

[19] Mohamed M.S., Mostafa M.M., Abdelfattah A.A. (2023). Prediction of side branch occlusion in bifurcational lesions during percutaneous coronary interventions by preprocedural coronary computed tomography using the CT bifurcation score. Postepy Kardiol. Interwencyjnej.

[20] Seo J.B., Shin D.H., Park K.W., Koo B.K., Gwon H.C., Jeong M.H., Seong I.W., Rha S.W., Yang J.Y., Park S.J. (2016). Predictors for Side Branch Failure during Provisional Strategy of Coronary Intervention for Bifurcation Lesions (from the Korean Bifurcation Registry). Am. J. Cardiol..

[21] Chen S.L., Zhang J.J., Ye F., Chen Y.D., Patel T., Kawajiri K., Lee M., Kwan T.W., Mintz G., Tan H.C. (2008). Study comparing the double kissing (DK) crush with classical crush for the treatment of coronary bifurcation lesions: The DKCRUSH-1 Bifurcation Study with drug-eluting stents. Eur. J. Clin. Investig..

[22] Arunothayaraj S., Behan M.W., Lefèvre T., Lassen J.F., Chieffo A., Stankovic G., Burzotta F., Pan M., Ferenc M., Hovasse T. (2023). Stepwise provisional versus systematic culotte for stenting of true coronary bifurcation lesions: Five-year follow-up of the multicentre randomised EBC TWO Trial. EuroIntervention.

[23] Chen S.L., Xu B., Han Y.L., Sheiban I., Zhang J.J., Ye F., Kwan T.W., Paiboon C., Zhou Y.J., Lv S.Z. (2013). Comparison of double kissing crush versus Culotte stenting for unprotected distal left main bifurcation lesions: Results from a multicenter, randomized, prospective DKCRUSH-III study. J. Am. Coll. Cardiol..

[24] Chen S.L., Xu B., Han Y.L., Sheiban I., Zhang J.J., Ye F., Kwan T.W., Paiboon C., Zhou Y.J., Lv S.Z. (2015). Clinical Outcome After DK Crush Versus Culotte Stenting of Distal Left Main Bifurcation Lesions: The 3-Year Follow-Up Results of the DKCRUSH-III Study. JACC Cardiovasc. Interv..

